# M. Chervin's Test for the Contagion of Cholera

**Published:** 1831

**Authors:** 


### M. Chervin's Test for the Contagion of Cholera.

M. Chervin is a very zealous, and certainly an able, party writer on yellow fever, and epidemic diseases in general. It was to be expected that cholera would arrest his attention; and it is a wonder that he did not repair to the scene of its devastations to enquire into its nature. He has, however addressed a letter to the Minister of the Interior, proposing a plan for testing the contagion (if it exist) of cholera morbus. He sets out by observing that, hitherto, the malady has set quarantines, cordons sanitaire, and lazarets, completely at defiance: and he fears, with some reason, that the French and English physicians who have repaired to Poland and Russia, will not be able to settle satisfactorily the question of contagion. He thinks the surest plan of ascertaining the point would be, to institute a series of experiments in some place where the epidemic influence does not yet prevail, and, consequently, where contagion, (if it exist at all) would be tested *per se*, and beyond the range of the original or more general cause of cholera.

He proposes some fixed point in the north-west of France for the trial. Having established a lazaretto, the next step is to collect some of those materials which are supposed to be most tenacious of contagion, and which are, at the same time, most unequivocally imbued with the secretions and excretions of patients labouring under the disease—for instance, shirts, cottons, bed-clothes, &c. worn or used by cholera patients. These are to be secured in the greatest state of impurity, hermetically sealed, and transmitted by a steam-boat, or other vehicle, with all possible expedition to the lazaretto, where people in health are to be clothed and surrounded with the infected materials. M. Chervin offers himself as the first individual for this experiment, and has no doubt that hundreds of his professional brethren will volunteer for the same service, in the cause of science and humanity. If these volunteers, or any of them, become affected with cholera, and if the disease remains confined to these individuals, they being subjected to every precaution which the quarantine laws can effect, we shall then have fair reason to conclude that the contagion of cholera not only exists, but is transmissible from place to place by means of clothes, &c. The experiment would also, he observes, determine pretty well the efficacy of quarantine and sanatory codes. On the other hand, if the experimenters remain free from the disease, notwithstanding all these exposures to the foci and nidi of contagion, he imagines that we may safely come to the conclusion that cholera morbus is not propagated by infected materials.

However well adapted, the foregoing experiment might be for ascertaining the important point in question, it is perfectly evident that the French Government will not adopt the proposal. If all France were savans and physicians, the plan might be put in execution. But the prejudices of the unlearned community must be respected; and we imagine that no small portion of this, the largest class in all countries, would protest against the measure, as a wanton, nay, an impious introduction of a pestilence which it was the duty of Government to exclude, by all possible means, from the territories over which they bore sway.

P.S. The Government has politely refused.

---

